# Deletion as novel variants in VPS13B gene in Cohen syndrome: Case series

**DOI:** 10.1515/tnsci-2022-0304

**Published:** 2023-09-01

**Authors:** Li Kang, Yixuan Ma, Peng Zhao

**Affiliations:** Department of Rehabilitation Medicine, Tianjin Children’s Hospital, 238 Longyan Road, Rui Jing District, Tianjin, 300134, China; Division of Sports Science and Physical Education, Tsinghua University, Beijing 100081, China

**Keywords:** Cohen syndrome, mutation, *VPS13B* gene

## Abstract

**Background:**

Cohen syndrome (OMIM No. # 216550) is a rare autosomal recessive disorder caused by homozygous mutation in the vacuolar protein sorting 13 homolog B (*VPS13B*) gene on chromosome 8q22.2. Clinical manifestations include hypermobile joints, microcephaly, intellectual disabilities, craniofacial and limb anomalies, and neutropenia. To date, more than 200 mutations of *VPS13B* have been reported in over 1,000 Cohen syndrome patients. This article reviews the clinical data of two cases of Cohen syndrome diagnosed by whole exome sequencing.

**Results:**

Both children visited for psychomotor retardation. Gene detection showed a mutation in 8q22.2, NM_017890.4 Intron38 c.6940+1G > T and heterozygotic deletion of exon 3-19 of the *VPS13B* gene (Case 1), and a mutation in 8q22.2, NM_017890.4 Intron38 c.6940+1G > T and 8q22, NM_017890.4 Exon56 c10334_10335del in the *VPS13B* gene (Case 2). The variation was predicted to be pathogenic by related software, and they have not been reported.

**Conclusion:**

Cohen syndrome should be considered in the differential diagnosis of any child with developmental retardation and neutropenia. The present study increases the mutation spectrum of the *VPS13B* gene and could be helpful in genetic diagnosis and genetic counseling in Cohen syndrome patients.

## 1 Introduction

Cohen syndrome (OMIM No. # 216550) is a rare autosomal recessive hereditary disease [1]. Cohen syndrome was first described by M. Michael Cohen Jr. in two affected siblings and one isolated case [2]. Mutation in the 13B vacuolar sorting protein gene, vacuolar protein sorting 13 homolog B (*VPS13B*) (8q22-8q23) gene, which is the only gene responsible for Cohen syndrome [3–5].

Cohen syndrome is a neurodevelopmental disease characterized by intellectual disabilities, short stature, hypotonia, microcephaly, musculoskeletal abnormalities, neutropenia, myopia, and facial abnormalities such as short philtrum and micrognathia [1,6–8]. The usual method of testing or screening for Cohen syndrome is whole exome sequencing [9]. To date, about 200 *VPS13B* mutations have been reported in nearly 1,000 cases of Cohen syndrome [10], of which about five cases have been genetically diagnosed in China [10,11]. Mutation includes missense/nonsense, regulatory, small deletions/insertions, and gross deletions/insertions, among which missense/nonsense variants are the most common [12]. The disease tends to be heterogeneous with a wide phenotypic variability. Distinct founder variants have been described in different areas.

In this study, we report the reviewed two novel pathogenic variants of *VPS13B* in patients from China and discuss the clinical and genetic variation characteristics of Cohen syndrome.

## 2 Case presentation

### 2.1 Case 1

**Figure 1:**
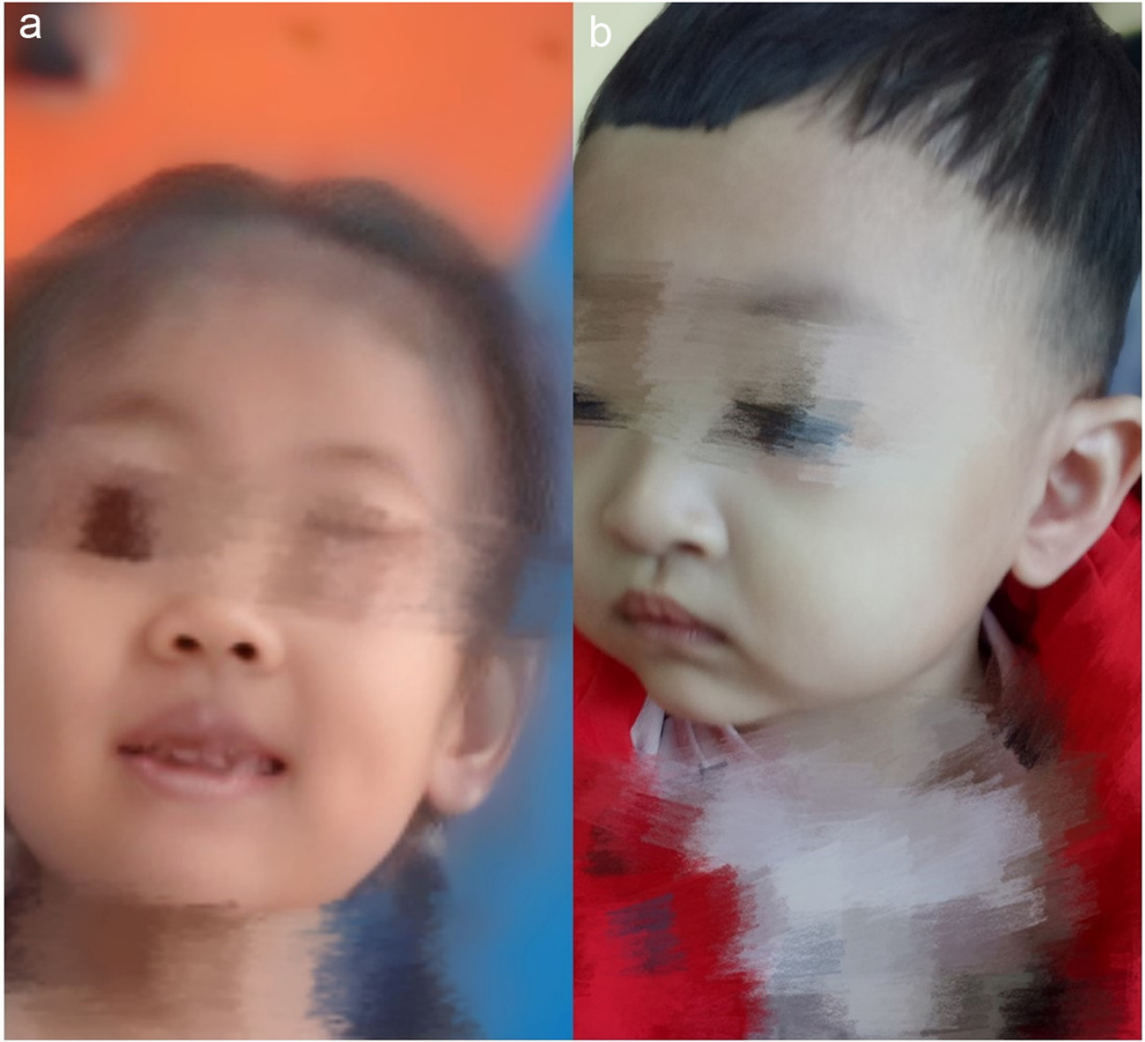
Characteristic facial features of case 1 (1a-left) and case 2 (1b-right).

**Figure 2:**
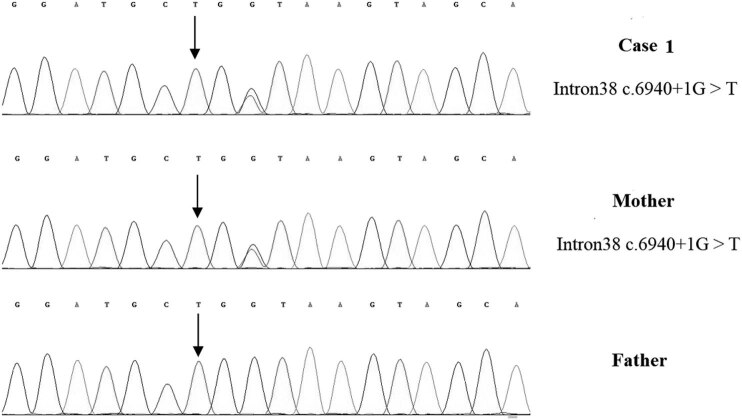
*VPS13B* Intron38 c.6940+1G > T heterozygous mutation in case 1, carried by the mother, but not detected by the father.

**Figure 3:**
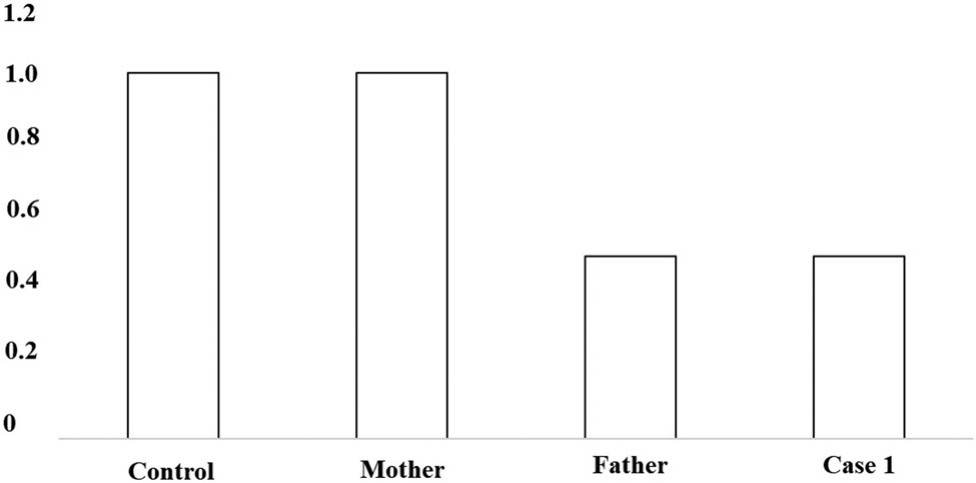
PCR verification: to the control, the ratio of copy number of *VPS13B* exon 3–19 in case 1 and her father was about 0.5, indicating that *VPS13B* had heterozygous deletion. To the control, the ratio of her mother’s copy number of *VPS13B* exon 3–19 was about 1, suggesting that her mother’s copy number was normal.

A 2-year-old girl was admitted to the hospital because she showed psychomotor developmental delay. She had a speech delay and typical facial characteristics include microcephaly (head circumference 42 cm), hypertelorism, thick eyebrows, thick bushy hair, low hairline, and micrognathia (Figure 1a). The Autistic Behavior Checklist (ABC) score was 61. Developmental quotient (DQ) from five domains (adaptive, social, language, gross, and fine motor) was assessed using Gesell Developmental Schedules; they all showed moderate retardation (40 ≤ DQ ≤ 54) [13]. The laboratory examination results were as follows: the routine blood test showed that the white blood cell count was 8.74 × 10^9^/L, the neutrophil count was 1.33 × 10^9^/L, and the neutrophil percentage was 15.2%. High-throughput whole-exome sequencing: an ethylene diamine tetraacetic acid (ETDA) blood sample was taken, and next-generation sampling revealed a mutation in 8q22.2, NM_017890.4 Intron38 c.6940+1G > T in the VPS13B gene (Figure 2). This mutation was graded in relevant mutation interpretation guidelines by the American College of Medical Genetics (ACMG) as pathogenic. Heterozygotic deletion of exon 3–19 of the *VPS13B* gene was also revealed. Semi-quantitative PCR for specific gene deletion results: the copy number of case 1 (exon 3–19 of *VPS13B* gene) and her father was 1, heterozygous deletion. The mother’s copy number is 2, which is normal, and the deletion is from the father (Figure 3).

### 2.2 Case 2

**Figure 4:**
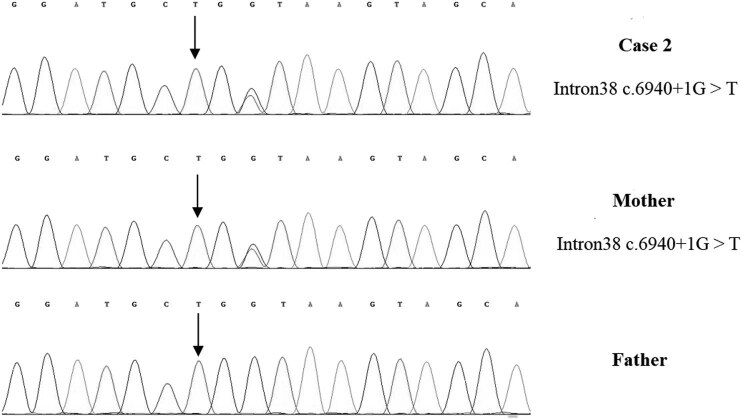
*VPS13B* Intron38 c.6940+1G > T heterozygous mutation in case 2, carried by the mother, but not detected by the father.

**Figure 5:**
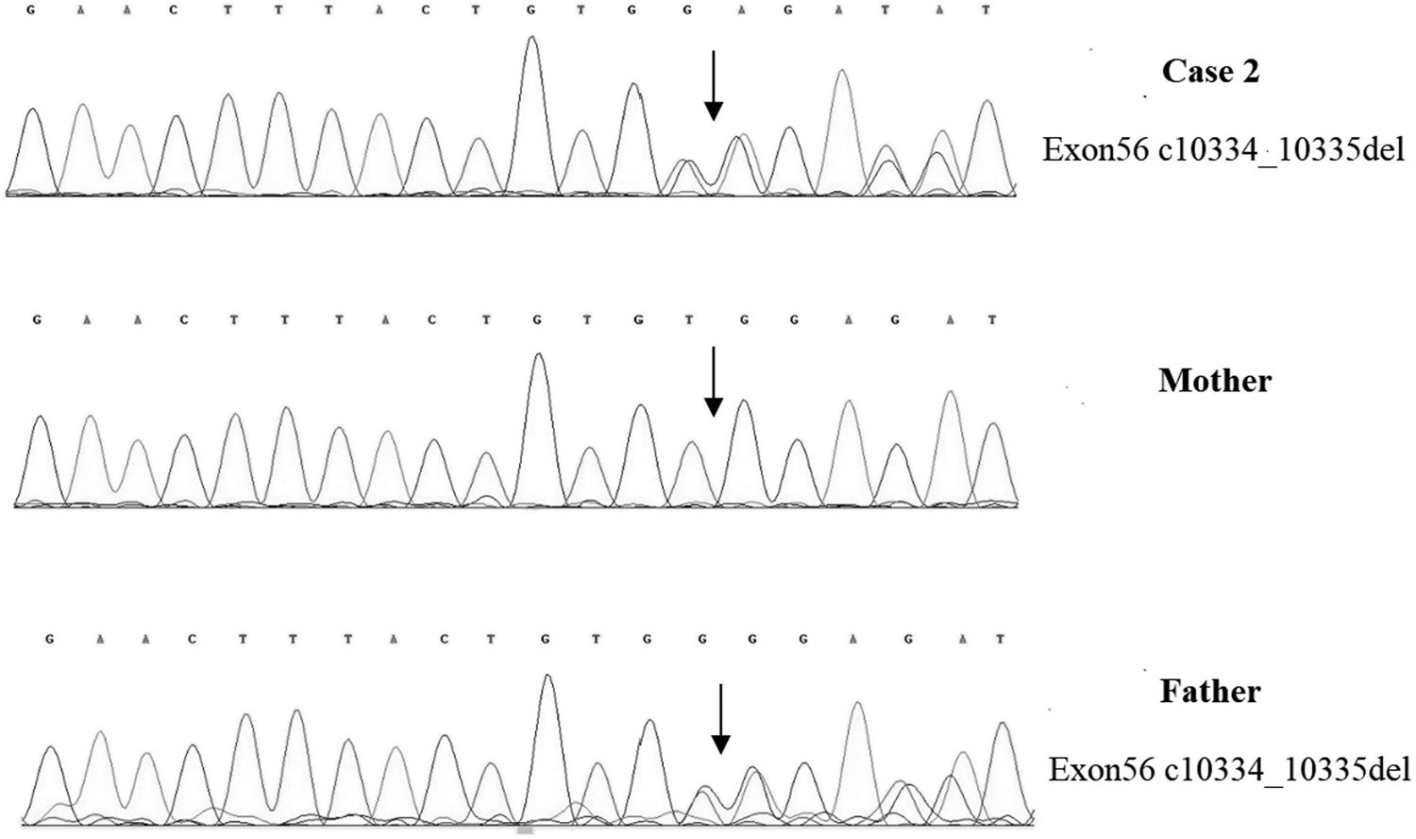
*VPS13B* Exon56 c10334_10335del heterozygous deletion in case 2, carried by the father, but not detected by the mother.

A 1-year-old boy was admitted to the hospital because of developmental retardation, microcephaly (head circumference 43 cm), and micrognathia (Figure 1b). The modified Checklist for Autism in Toddlers (M-chat) score was 28. DQ from all five domains showed severe retardation (25 ≤ DQ ≤ 39) [13]. The laboratory examination results were as follows: the routine blood test showed that the white blood cell count was 7.60 × 10^9^/L, the neutrophil count was 1.26 × 10^9^/L, and the percentage of neutrophils was 16.5%. High-throughput whole-exome sequencing: an ETDA blood sample was taken, and using next-generation sampling, the results came out revealing a mutation in 8q22, NM_017890.4 Intron38 c.6940+1G > T (Figure 4) and 8q22, NM_017890.4 Exon56 c10334_10335del in the *VPS13B* gene (Figure 5). These two mutations were graded in relevant mutation interpretation guidelines by the ACMG as pathogenic.

The clinical manifestations, relevant check results, and high-throughput whole-exome sequencing of the two children are summarized in Table 1. The pathogenicity of the mutations evaluated according to ACMG guidelines is detailed in Table 2.

**Table 1:** Detailed clinical manifestations, relevant check results, and high-throughput whole-exome sequencing

Case number	1	2
Gender (girl/boy)	Girl	Boy
Age (years)	2	1
Similar affected family member	No	No
**Anthropometry**		
Height (cm)	90	78
Weight (kg)	11.6	10.6
Head circumference (cm)	42	43
Pregnancy history	Gravida 1 para 1	Gravida 1 para 1
Onset	About 1 year old	About 7 months
Clinical manifestations	Hypertelorism	Micrognathia
	Thick eyebrows	Developmental retardation
	Thick bushy hair	
	Low hairline	
	Micrognathia	
	Developmental retardation	
**Radiological reports**		
Cranial magnetic functional evaluation	A widened brain gap	Cerebellar dysplasia
ECG	Sinus rhythm, right deviation of electrical axis, QR type of AVR, and no abnormality of ST segment	Normal
Autism assessment	ABC score was 61	M-chat score was 28
Gesell scale	Severe developmental retardation in five energy regions	Severe developmental retardation in five energy regions
**Laboratory investigations**		
The percentage of neutrophils (%)	25.0	20.5
**Variant**		
High-throughput whole-exome sequencing	8q22.2, NM_017890.4 Intron38 c.6940+1G > T in the *VPS13B* gene; p.?. And heterozygotic deletion of exon 3–19 of *VPS13B* gene	8q22, NM_017890.4 Intron38 c.6940+1G > T (Figure 3) and 8q22, NM_017890.4 Exon56 c10334_10335del in the *VPS13B* gene

**Table 2:** Pathogenicity evaluation

	ACMG evidence
**Case 1**	
c.6940+1G > T	**PVS1**: this variant affects the GT donor site of intron 38 and has been proved to cause exon 38 skip at mRNA level (PMID: 33959574)
	**PM3_strong**: the variant was observed in trans with other pathogenic variants in several cases (PMID: 33959574, 33025479, 30138938, and in the current report)
	**PS3**: functional analysis has been conducted by Guiyu Lou et al. and Liangshan Li et al. (PMID: 33959574, 33025479)
E3–19 del	**PVS1**: this variant causes loss of a long segment of the whole gene
	**PM2**: this variant is very rare in common population
	**PM3**: the variant was observed in trans with other pathogenic variants in our case
**Case 2**	
c.6940+1G > T	Same as above
c.10334_10335del	**PVS1**: this variant causes frame shift and truncation of the 3′ terminal 7 exons
	**PM2**: this variant is very rare in common population and has not yet been collected in gnomAD database
	**PM3**: the variant was observed in trans with other pathogenic variants in our case


**Ethical approval:** The research related to human use has been complied with all the relevant national regulations, institutional policies and in accordance with the tenets of the Helsinki Declaration, and has been approved by the authors’ institutional review board or equivalent committee.
**Informed consent:** Informed consent has been obtained from all individuals included in this study.

## 3 Discussion

We reported two novel compound heterozygous variants in the *VPS13B* gene inherited from non-inbred parents. Although this variant (cases 1 and 2) 8q22, NM_017890.4 Intron38 c.6940+1G > T in the *VPS13B* gene had been detected in patients with Cohen syndrome [7], the deletion of exon 3–19 and Exon56 c10334_10335del of *VPS13B* gene could constitute complex heterozygous mutations that cause Cohen syndrome with mutation in Intron38 c.6940+1G > T. The deletion range of these two fragments, which has not been reported in the literature, is a newly discovered variation that extends the spectrum of genes associated with Cohen syndrome.

The most significant manifestations of the cases reported in this article are developmental retardation and neutropenia. This result is consistent with the clinical features reported in other literature [14,15]. The possible mechanism of *VPS13B* causing neutropenia is related to increased neutrophil apoptosis and decreased SerpinB1 expression, which is a critical component of neutrophil survival [15]. In addition, in Cohen syndrome neutrophils, the *SERPINB1* gene, which encodes a critical component of neutrophil survival, is significantly reduced, leading to excessive apoptosis of neutrophils, which is a possible cause of neutropenia [16]. The probable cause of developmental retardation is that *VPS13B* gene mutation leads to the reduction of coding protein which is a Golgi-enhanced scaffold protein that helps to maintain the structure and function of the Golgi complex. It has been reported that *VPS13B* regulates functional neuronal networks [7].

Cohen syndrome is a clinically heterogeneous disease with extensive and variable clinical manifestations, such as myopia, retinal dystrophy, and heart disease [14,15]. Unlike other cases, our patient did not present any ophthalmologic or cardiologic abnormality [17]. Therefore, there are some difficulties in the early diagnosis of the disease. And for clinical, there is no unified diagnostic standard for Cohen syndrome. The diagnosis can be determined mainly based on clinical manifestations, including obesity, hypotonia, mental deficiency, and facial, oral, ocular and limb anomalies: leukopenia, especially neutropenia. The pathogenic variation of the *VPS13B* gene was detected by molecular genetics [1,6,15]. According to previous research reports, there is no clear correlation between severity of the disorder and variant [1,12]. Further research is needed on the relationship between genotype and phenotype of Cohen syndrome.

Management of Cohen syndrome includes regular monitoring, rehabilitation, and genetic counseling. Ophthalmology should regularly evaluate visual acuity, refractive errors, and retinal dystrophy. Routine blood tests are performed to assess neutropenia. More frequent monitoring may be required for individuals with low absolute neutrophil count or recurring infection. Growth and weight gain also should be monitored. Treatment for patients with Cohen syndrome is symptomatic, including early physical, occupational, and speech therapy to address developmental retardation, hypotonia, joint hyperactivity, and granulocyte colony-stimulating factor to improve neutropenia [15].

## 4 Conclusion

We found two novel compound heterozygous variants in the *VPS13B* gene that can induce Cohen syndrome. In clinical diagnosis, if the children had special facial features, joint hyperactivity, neutropenia, and multi-system involvement, great attention should be paid to the possibility of genetic diseases. Therefore, genetic testing is significant for early diagnosis.
